# Convergence in Coevolved Systems: A Two-Axis Filter for Biomimetic Transferability

**DOI:** 10.3390/biomimetics11070446

**Published:** 2026-06-26

**Authors:** Ozren Polašek

**Affiliations:** 1 Croatian Science Foundation, 10000 Zagreb, Croatia; ozren.polasek@hrzz.hr or ozren.polasek@mefst.hr; 2 School of Medicine, University of Split, 21000 Split, Croatia

**Keywords:** evolution, convergence, biomimetics, parasite, dyad

## Abstract

Biomimetics often treats convergent evolution as the strongest sign that a biological solution is general. That inference is safest when the constraint does not counter-adapt. Hosts do. In coevolved host-interface systems, recurrence alone cannot tell us whether a solution is translatable. Biomimetic transferability depends first on two axes: conservation of the host target and versatility of the attacking lineage. A conserved target behaves, for translational purposes, like a biochemical constraint, a broad-host parasite has already tested its mechanism across biological variation. The window narrows when the target is taxonomically local, or when the mechanism has become a private molecular conversation inside a narrow dyad. Haematophagous feeders, intracellular protozoans, specialist helminths, and polydnavirus-bearing parasitoid wasps therefore do not offer the same kind of biomimetic object. Some yield molecules, some vulnerability maps, some contextual principles, and some only architecture or analogy. The point is not to mine coevolved systems less, but to stop mistaking coevolutionary success for biomimetic portability.

## 1. Introduction

Convergence is one of evolutionary biology’s most persuasive signals [1]. When distant lineages repeatedly arrive at similar forms, functions, or mechanisms, the recurrence appears to uncover a constraint [2]. The problem was met more than once. The solution was found more than once. The solution may therefore be general rather than accidental. Recurrent cases include camera eyes, streamlined aquatic bodies, powered flight, echolocation, C4 photosynthesis, antifreeze proteins, venoms, and immune-evasion systems [3,4,5,6]. Biomimetics inherits the logic [7,8,9]. A recurrent evolved solution is not merely explained. It is mined.

The inference is legitimate in some domains and dangerous in others [9,10]. The difference lies less in recurrence itself than in the kind of constraint that produced it. Light imposes stable optical demands. Water imposes hydrodynamic demands. Fracture mechanics imposes structural limits. These constraints are not simple, but they are not adapting adversaries [9,11].

Hosts are. A host is an evolving partner, with its own phylogenetic history, physiological architecture, immune repertoire, tissue states, microbiome, and counter-adaptations to a parasitic organism [12]. Convergence in such systems does not necessarily mean that the same mechanism has been rediscovered [11]. It may only mean that different organisms repeatedly faced a similar strategic problem and solved it through different, lineage-bound routes [12]. This distinction matters because portability is not the same as adaptation [13]. The more deeply a mechanism has been shaped inside a narrow reciprocal interaction, the more likely it is to depend on partner-specific biology [14]. Evolutionary success inside the dyad may predict translational failure outside it [12]. The question is therefore not whether convergence occurred. It is whether the recurrence sits in the right part of biological space to make the solution translatable.

## 2. The Biomimetic Window

The proposal is a two-axis filter for biomimetic portability in coevolved host-interface systems. The first axis is conservation of the host target. The second is versatility of the attacking lineage. These axes are related, but not interchangeable.

### 2.1. Target Conservation

Target conservation asks whether the attacked host structure, receptor, pathway, tissue process, or physiological system is shared across the intended translational space. For translational purposes, a conserved target behaves more like a biochemical constraint than a private host feature. Haemostasis, complement activation, vascular tone, pain signalling, epithelial injury responses, and core inflammatory pathways are not invariant, but they are conserved enough across vertebrates that repeated attack on them can expose portable pharmacology [15,16,17,18].

### 2.2. Parasite Versatility

Parasite versatility asks whether the attacking lineage has been selected across a broad range of hosts rather than within a single coevolved dyad. A versatile parasite has had to make its solution work in more than one host setting. The mechanism must operate during real infection, in the relevant tissue state, and across repeated natural encounters [19]. Most importantly, the same mechanism must travel across hosts, not merely the same broad outcome. A specialist parasite may instead become highly effective inside one narrow corridor: stronger locally, but less exportable [14]. Throughout this article, parasite is used functionally rather than taxonomically, to include host-interface feeders and parasitoid systems.

### 2.3. Functional Versus Mechanistic Convergence

Host-interface systems often converge functionally before they converge mechanistically [19]. Functional convergence is the repeated emergence of an effect: immune evasion, coagulation blockade, intracellular persistence, tissue repair modulation, pain suppression, and tolerance induction. Mechanistic convergence is narrower and rarer: recurrence at the level of target, causal route, molecular structure, pathway architecture, or delivery logic. Biomimetics often fails at precisely this distinction. It treats repeated immune regulation as evidence for transferable immunomodulators, repeated intracellular persistence as evidence for reusable cellular-control machinery, and repeated host manipulation as evidence for general design principles. None of these inferences is secure by recurrence alone. Functional recurrence names the problem. It does not identify the transferable mechanism.

Multiple parasite lineages often converge on the same pathway node, namely, IL-10 induction, complement inhibition, and T-cell expansion, but arriving there through different molecular routes [20,21,22]. In such cases, the filter’s utility is to ask whether the node itself is a conserved, transferable target, or merely a redundant endpoint reached by lineage-specific mechanisms. If the node is conserved, transfer is plausible. If different lineages reach it by different causal chains, the mechanism itself is less likely to be portable.

### 2.4. Where the Window Opens and Closes

The biomimetic window opens where the host target is conserved and the attacking lineage is versatile enough to engage that target across biological diversity. This is the high-yield region. Haematophagy is the canonical case: vertebrate haemostatic, vascular, complement, inflammatory, and sensory systems are deeply conserved [15,16,23,24], while many blood-feeding lineages have been selected to manipulate them across repeated host encounters [25]. Even here, evolutionary promise does not guarantee clinical utility. Toxicity, manufacturability, delivery, immunogenicity, pharmacokinetics, and intellectual-property constraints can still defeat translation.

The window closes in two directions. It closes from below when the target is too local: the problem solved by the organism is real but taxonomically narrow, ecologically parochial, or irrelevant to human biology. It closes from above when the mechanism is too host-tuned: the problem is important, but the solution has become a private molecular conversation between parasite and host. In specific dyads, parasite load can itself become a sustained selective pressure, steering host biology away from the ancestral or broadly shared state [11]. The interaction then becomes doubly local: the parasite is tuned to the host, and the host has been partly reshaped by the parasite [14]. Transferability falls from both sides. The first failure is insufficient scale. The second is coevolutionary overfitting. It signifies the situation in which the parasite’s effective host range collapses to a single coevolved partner, and the host shows reciprocal signatures of adaptation through local shifts in immune repertoire, counter-evolved metabolic or physical defences [11,14,26]. The solution has become too specific to solve a general problem.

The framework is therefore non-monotonic. More coevolution is not always better. Less specificity is not always better. Biomimetic value peaks where the biological problem is large enough to matter, but the mechanism has not become locked to its original interaction.

### 2.5. Wider Coevolutionary Scope

Coevolutionary biomimetics is often tied to host–parasite arms races, but some of its most productive applications come from cooperative and predator–prey systems instead. A clear case is the coral–alga mutualism; “bionic corals” can grow microalgae at densities roughly a hundred times higher than conventional culture [27]. A second example draws on the legume–rhizobia symbiosis, an example of a cooperative relationship that synthetic biologists are now attempting to transplant into cereals like rice and wheat [28,29]. Predator–prey coevolution contributes too: in the bat–moth acoustic arms race, moths evolved wing scales that act as an ultrathin sound-absorbing metamaterial to evade bat sonar, now copied to design thin, broadband acoustic panels [30]. The common thread is that any reciprocal evolutionary pressure, not just antagonism between a host and its parasite, can drive the refined, problem-solving design that engineers find worth abstracting and translating. These cases show that coevolution functions as a broad design engine across the full spectrum of ecological interactions [31], with host–parasite systems representing just one corner of a much larger space.

## 3. Parasite–Host-Interface Systems as a Two-Axis Test Case

The two axes define a simple but informative test: target conservation is set against parasite versatility. The examples below are not a catalogue. They are a stress test, chosen to populate the two-axis space with enough biological diversity to show why recurrence alone can mislead. Other host-interface systems, such as ectoparasitoid Hymenoptera outside the polydnavirus-bearing clade, strepsipteran parasitoids, monogenean flatworms, and apicomplexan tissue cyst-formers, could be placed in the same space. The four groups used here span the range with minimal redundancy, since they all converge on host manipulation (Table 1). They do not, however, yield the same translational object.

The placement of parasite classes in Table 1 reflects two axes. For target conservation, dimensions that matter include phylogenetic breadth of the target structure, sequence and structural homology of the interface, whether redundant pathways bypass the target, and whether the host shows evidence of rapid evolution or polymorphism at the attack point. For parasite versatility, dimensions include hosts where the life cycle completes naturally (not experimental infection), the phylogenetic spread of those hosts, whether distinct lineages use the same mechanism to infect them (not merely achieve the same outcome), and the physiological range those mechanisms tolerate.

### 3.1. Haematophagous Feeders: Conserved Targets, Broad Biological Testing

Haematophagous feeders occupy the clearest high-yield region. Leeches, ticks, mosquitoes, sandflies, triatomines, and other mammalian-skin blood-feeders must acquire blood despite haemostasis, platelet activation, vasoconstriction, complement, inflammation, pain, itch, and tissue repair [32,33,34]. These host systems vary across vertebrates, but not enough to lose translational relevance. They behave as conserved interface machinery.

Many haematophagous lineages also operate across broad or structured host ranges [35,36]. Their molecules must work quickly, locally, and repeatedly in different host backgrounds. This makes their salivary and secreted repertoires unusually plausible sources of portable pharmacology [25,37]. The anticoagulant and anti-haemostatic agents are the canonical successes. A molecule may be found in one leech or tick lineage; its broader significance is strengthened because many independent blood-feeding lineages face the same conserved problem. Convergence is the evidentiary amplifier, not necessarily the discovery engine.

The second sub-region is salivary immunomodulation [33,34,38]. It shares the favourable axes: conserved host targets, repeated interface attack, and exposure to vertebrate variation. Yet its clinical record is less mature, still in the candidate stage [39]. This should not be read as a failure of the underlying biology. It may reflect research history, candidate selection, delivery difficulty, and weaker prospective filtering. Here, convergence should function less as retrospective validation than as a forward discovery filter. The transferable object is therefore not the ecological habit, but the conserved host physiology that blood-feeding repeatedly exposes.

### 3.2. Intracellular Protozoan Host-Cell Manipulators: Conserved Vulnerabilities, Divergent Machinery

Intracellular protozoan parasites occupy a more ambiguous region [40]. *Plasmodium*, *Toxoplasma*, *Leishmania*, *Trypanosoma cruzi*, and related systems repeatedly expose host vulnerabilities: cell entry, intracellular survival, phagolysosomal maturation, antigen presentation, interferon signalling, immune memory, cell death, nutrient acquisition, and tissue persistence [41,42]. Many of these host processes are conserved and medically important. The target axis is therefore often favourable.

The mechanism axis is less favourable. Protozoan parasites often converge strategically while diverging causally. Antigenic variation recurs, but its architectures differ [43]. Intracellular survival recurs, but vacuole remodelling, cytosolic escape, host-cell modification, immune-signalling interference, and surface variation are frequently lineage-specific. The same host vulnerability is exploited, but the parasite machinery may not travel.

This is not a low-value category. It is a different value category. Protozoan host-cell manipulators are more likely to yield vulnerability maps, host-process bottlenecks, vaccine strategies, drug susceptibilities, and pathway-level insight than directly portable parasite-derived therapeutics [44,45]. The window is open for target discovery, but narrower for direct molecular borrowing.

### 3.3. Specialist Helminths: Strong Strategic Convergence, Weak Portability

Specialist helminths are the seductive failure case. Their biology seems built for biomimetic optimism. Chronic helminth infection is repeatedly associated with regulatory T-cell expansion, IL-10-rich environments, modified type-2 immunity, alternatively activated macrophages, eosinophil modulation, epithelial repair, altered antigen presentation, and dampening of inflammatory pathology [46]. The functional convergence is unmistakable [47,48].

The translational record has not matched the conceptual promise [49,50]. Helminth therapy and helminth-derived immunomodulation have produced important biology and scattered candidates, but not the broad therapeutic harvest implied by the rhetoric of parasite-induced tolerance. *Trichuris suis* is emblematic. The pig parasite was trialled in human Crohn’s disease on the expectation that immune-regulatory effects would generalise. The TRUST-I and TRUST-II trials did not support that expectation [51]. The result does not show that helminth immunomodulation is futile [52]. It shows that functional convergence was overread.

The two-axis model explains why. Helminths often target immune systems conserved in broad outline but variable in regulatory detail. Many have narrow host ranges, complex life cycles, tissue-stage dependencies, and long histories of host-specific accommodation [53]. Their mechanisms may be potent and still dyad-tuned. The target is important; the mechanism may be too embedded in host context to travel cleanly. Specialist helminths may yield immune principles, context-dependent mechanisms, or carefully selected molecules acting on conserved pathways. They should not be treated as libraries of ready-made human immunotherapies [52].

### 3.4. Polydnavirus-Bearing Parasitoid Wasps: Outside the Molecular Window

Polydnavirus-bearing parasitoid wasps sit at the deep boundary. Braconid and ichneumonid wasps have domesticated viral systems integrated into their germline [54,55], produced in reproductive tissues, and delivered into insect hosts during oviposition. These particles do not function as ordinary replicating viruses in the parasitised host. They deliver genes that suppress immunity, alter development, and preserve the host as a living resource for parasitoid offspring. As molecular sources for human immunomodulation, these systems are poor candidates. The human target is weakly conserved or absent, and the attacking system is deeply dyad-locked. They lie outside the molecular biomimetic window.

A cautious architectural reading remains possible, but only under strict conditions. A deep specialist system is architecturally useful only if it solves a generalisable design problem named before the analogy is made. On that test, most attractive features of polydnavirus biology, such as modularity, replication control, and host-state manipulation, are too underspecified to support a direct prospective transfer. Two features survive more cleanly: germline-integrated production of non-replicating particles for delivery into another organism [54,56], and temporal separation of vector production from host deployment. These map onto recognisable engineering problems in delivery and containment. They do not rescue the system as a molecular source.

## 4. Prospective Operationalisation

**Figure 1:**
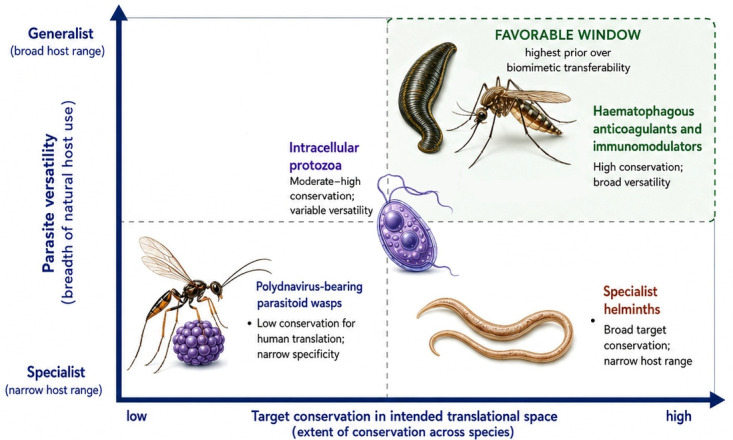
The two-axis filter for biomimetic transferability in coevolved host–interface systems. The horizontal axis represents target conservation (low to high); the vertical axis, parasite versatility (specialist to generalist). The shaded region (upper right) marks the favourable window where both axes score high. Four parasite classes are positioned: haematophagous anticoagulants and immunomodulators occupy the window or its edge (high conservation, broad versatility); intracellular protozoa occupy the centre-right (moderate-to-high conservation, variable versatility); specialist helminths fall to the right but low on the versatility axis (broad target but narrow host range); polydnavirus-bearing wasps fall outside the window (low conservation for human translation, narrow specificity).

A framework that only redescribes past success and failure is weak [7,57]. Minimal prospective assessment requires four questions. These questions do not produce a score, as they cannot eliminate uncertainty in coevolved systems. Instead, they yield a reasoning protocol: a structured way to ask whether the claimed transfer is biologically plausible given what we know about target conservation and parasite versatility. The output is a prior, not a prediction (Figure 1).

First, what is the host target? The answer must be specific enough to distinguish a conserved physiological system from a vague endpoint. Immune modulation is not a target [58]. Stronger candidates name a tractable process, mediator, or interface: IL-10 induction, TGF-β signalling, complement blockade, platelet aggregation, epithelial alarmin release, keratinocyte state change, wound-edge migration, macrophage polarisation, kinin signalling, nociceptor modulation, microbiome restructuring, or defined bioactive metabolites.

Second, how conserved is that target across the intended translational space? This can be assessed through comparative physiology, phylogenetic conservation, receptor homology, pathway architecture, functional redundancy, and evidence of rapid evolution or balancing selection [11]. More broadly, prospective biomimetic inference depends on placing organisms and traits in the correct phylogenetic frame; without that, recurrence, host range, and apparent similarity can easily be misread [10,59].

Third, how broad is the attacking lineage’s effective host range? Effective breadth is not the number of recorded hosts [19,60]. It includes completion of the life cycle, repeated natural exposure, activity across host states, tissue tropism, and whether the same mechanism acts across hosts. Opportunistic infection and experimental permissiveness should not be confused with evolutionary filtering across host diversity.

Fourth, what level of transfer is being claimed: molecule, pathway, vulnerability, context, architecture, or analogy? The claim must be explicit ex ante [7]. If the transfer object changes after failure, from molecule to architecture, from drug to inspiration, then the framework has become post hoc rhetoric. This question separates a disciplined biomimetic prior from retrospective salvage. The methodological move parallels transferable-unit specification in the ex natura protocol developed for constraint-stable biomimetics [9]. The parasitic case requires different variables but the same discipline. The filter governs source selection; downstream design-stage methodologies then govern how a chosen principle is engineered into an artefact [61].

These questions do not produce a single score. They produce a disciplined prior. More importantly, they force biomimetic projects to state what convergence is being taken as evidence for: molecular portability, target generality, system architecture, or biological interest. A system scoring high on both axes (conserved target, broad versatility) enters a favourable region. A system scoring low on either (novel target, narrow host range) does not merit abstraction at that level. Most systems fall in between, yielding a mixed prior that guides whether further investment is warranted and at what level of detail the mechanism can be expected to transfer.

### Retrospective Application: Trichuris Suis Infection in Crohn’s Disease

The framework asks four questions of the *Trichuris suis* immunomodulation case, trialled in TRUST-I and TRUST-II [51,62,63,64]. (1) What is the target? IL-10 production and regulatory T-cell expansion in the gut mucosa-a real, measurable outcome. (2) How conserved? IL-10 and Treg differentiation occur across vertebrates, but the regulatory landscape is host-variable and subject to polymorphism at immune-receptor loci. The target exists broadly but operates differently in each host. (3) How versatile is *T. suis*? It is a specialist with a narrow effective range, yielding low versatility. (4) What transfer is claimed? A molecular or biological principle extracted from *T. suis* that would induce immune tolerance in human patients. With a moderately conserved target and low parasite versatility, the framework suggests moderate-to-low transferability.

## 5. Consequences for Evolutionary and Biomimetic Inference

For evolutionary inference, convergence should not be treated as one evidentiary currency. Recurrence under stable physical constraint and recurrence under reciprocal biological interaction do not carry the same meaning. The first may indicate a narrow design space. The second may indicate shared strategic pressure, local adaptation, or convergent exploitation of conserved targets. The level of recurrence must be named [2,65].

For biomimetics, the distinction is more severe. A repeated evolved solution is not automatically a transferable solution [9,10]. Portability depends on the relation between the target and the evolutionary test the mechanism has already survived. Biomimetic programmes should ask not only whether nature has solved a problem, but what kind of problem was solved, against what target, across what range of biological variation, and at what level the solution can travel.

This reframes convergence from a general invitation into a structured filter. It does not reduce the value of nature as a source of design. It protects that value from indiscriminate extraction. The point is not to mine less. It is to mine at the right level. Repeated solutions do not guarantee portability. In coevolved systems, convergence remains one of evolution’s strongest signals, but it is not self-interpreting. The task for evolutionary biomimetics is to apply the filter prospectively, before success has made the answer obvious and failure has made the analogy decorative.

## Figures and Tables

**Table 1:** Parasite classes in the two-axis space.

Parasite Class	Target Conservation	Effective Host Range	Convergence Signal	Expected Output
Haematophagous feeders (anticoagulant sub-region)	High	Often broad or structured	Causal convergence on conserved haemostatic systems	Molecules; established pharmacological leads; convergence as evidentiary amplifier
Haematophagous feeders (salivary immunomodulator sub-region)	High	Often broad or structured	Causal convergence on conserved immune-regulatory targets	Molecules under prospective filtering; convergence as forward-looking discovery filter
Intracellular protozoan host-cell manipulators	Moderate to high	Variable	Strategic recurrence around conserved vulnerabilities with divergent mechanisms	Vulnerability mapping; host targets; pathway insight
Specialist helminths	Broad immune themes conserved; regulatory detail variable	Often narrow or host-state-restricted	Strong strategic convergence, weaker molecular portability	Context-dependent immune principles; cautious molecule mining
Polydnavirus-bearing parasitoid wasps	Low for human molecular translation	Narrow and dyad-tuned	Integrated host-manipulation architecture	Delivery logic and containment, restricted to design problems specified in advance

## Data Availability

No data were generated for this study.
